# A Retrospective Study of Cytology, High-Risk HPV, and Colposcopy Results of Vaginal Intraepithelial Neoplasia Patients

**DOI:** 10.1155/2018/5894801

**Published:** 2018-05-08

**Authors:** Qing Cong, Yu Song, Qing Wang, Hongwei Zhang, Shujun Gao, Long Sui

**Affiliations:** ^1^Obstetrics and Gynecology Hospital of Fudan University, Shanghai, China; ^2^Shanghai Key Laboratory of Female Reproductive Endocrine Related Diseases, Shanghai, China; ^3^Shanghai Medical Center of Key Programs for Female Reproductive Diseases, Shanghai, China

## Abstract

There is currently no large sample data of cytology, high-risk human papillomavirus (hrHPV), and colposcopy results of vaginal intraepithelial neoplasia (VaIN) in women who underwent hysterectomy and those who did not. We aim to explore the values of cytology, hrHPV, and colposcopy reports in detecting VaIN. A retrospective study of women diagnosed with VaIN by colposcopy-directed biopsy was performed at the Obstetrics and Gynecology Hospital of Fudan University, China, between January 1, 2014, and December 31, 2014. A total of 529 cases of VaIN were diagnosed, including 16.1% VaIN2/3 and 83.9% VaIN1. The ratio of VaIN2/3 in VaIN among patients after hysterectomy and with an intact uterus was 35.1% and 12.0%, respectively. The sensitivity of cytology for VaIN2/3 in only, concomitant, and posthysterectomy VaIN was 42.1%, 80.0%, and 80.8%, respectively. The sensitivity of hrHPV and cytology/hrHPV cotesting for VaIN2/3 in patients with an intact uterus versus those after hysterectomy was 93.5% versus 92.3% and 92.0% versus 100.0%, respectively. Notably, 13.3% of the patients with VaIN and 9.7% of the patients with VaIN2/3 underwent hysterectomy for noncervical diseases. The sensitivity of cytology and hrHPV for VaIN is noninferior to that of CIN2+, and thus these methods can help in the early detection of VaIN effectively.

## 1. Introduction

Vaginal cancer is a human papillomavirus- (HPV-) associated gynecologic disease, accounting for approximately 1% to 4% of cancers of the female genital tract. High-grade squamous intraepithelial lesion (HSIL) or vaginal intraepithelial neoplasia (VaIN) grade 2/3 is a precancerous lesion analogous to HSIL/cervical intraepithelial neoplasia (CIN) grade 2/3 [1]. Low-grade squamous intraepithelial lesion (LSIL) or VaIN 1 is a benign manifestation of HPV infection. The natural history of VaIN is thought to be similar to that of CIN. In the past, VaIN was rarer than vaginal invasive cancer because it was frequently underdiagnosed [2, 3]. The reported incidence rate of vaginal cancer is 0.4 to 0.6 per 100,000 women, while the incidence of VaIN is 0.2–0.3 per 100,000 women [4–6]. The reported frequencies were 0.5% of all neoplastic lower genital tract lesions [7].

Over recent decades, the diagnosis of vaginal intraepithelial neoplasia (VaIN) has increased steadily as a result of widespread application of cytology/high-risk human papilloma virus (hrHPV) cotesting and colposcopy in cervical cancer screening. In the colposcopy clinic of the largest obstetrics and gynecology tertiary teaching hospital in China, the detection rate of VaIN in all lower genital tract intraepithelial lesions was 11% (1,923/16,732) on average, with an increasing trend from 2013 to 2015 [8]. However, few studies have investigated the cytology, hrHPV, and colposcopy results in VaIN. The number of VaIN cases included in currently available studies was limited to 6 to 132 cases, and most focused on posthysterectomy patients [5, 9–14], because current cervical cancer screening guidelines recommend that women who have had cervical precancer or invasive cervical cancer undergo continued surveillance testing for at least 20 years after treatment [15]. Besides, many women after total hysterectomy for benign diseases undergo vaginal cytology and/or hrHPV tests, and clinicians are faced with dilemmas of managing their abnormal results [16]. Should women after total hysterectomy for benign diseases be referred to colposcopy or just leave it? The meaning or value of these abnormal results needs to be investigated.

Up till now, there is limited data on cytology, hrHPV, and colposcopy of VaIN in women who underwent hysterectomy and those who did not. In our hospital, the largest obstetrics and gynecology tertiary teaching hospital in China, women, including those who underwent hysterectomy and those who did not, undergo regular cytology and/or hrHPV testing; those with abnormal screening reports are referred to colposcopy. On this basis, a large retrospective study of VaIN patients was performed to explore the values of cytology, hrHPV, and colposcopy in detecting VaIN, which might help understand clinical characterization of VaIN, including distribution of VaIN1 and VaIN2/3, cytology/hrHPV sensitivity, and indications of previous hysterectomy of VaIN. To the best of our knowledge, this is the largest retrospective study of cytology and hrHPV results in VaIN to date.

## 2. Methods

All women diagnosed with VaIN by colposcopy-directed biopsy between January 1, 2014, and December 31, 2014, at the Obstetrics and Gynecology Hospital of Fudan University were included. VaIN was histologically diagnosed by two independent gynecologic pathologists. Women with abnormal cytology but normal histological diagnosis were excluded. Approval was obtained from the institutional review board of the Obstetrics and Gynecology Hospital of Fudan University before data extraction was performed and consent to research signed. All available data including demographics, history, histological information, cytology, and hrHPV testing results were recorded. Bethesda System terminology was used for reporting cytology results [17]. In 529 patients with VaIN, 517 had complete medical history data. Their original composition of vaginal, cervical, and vulvar lesions was listed when history of cervical or vulvar lesions was considered (Table 1).

There were 222 patients with only VaIN, because 41 of 263 patients with only VaIN included in Table 3 had a history of cervical or vulvar conization or laser ablation. In total, 42.9% of the patients had only VaIN and 57.1% had concomitant cervical or vulvar lesions. And 15.9% of the cases were VaIN2/3 and 84.1% were VaIN1.

Regular cytology and/or hrHPV screening are performed in all women after hysterectomy. Women with abnormal cytology or hrHPV reports were referred to colposcopy in our hospital. Women with a prior hysterectomy performed for cervical lesions were routinely referred to colposcopy at least once. Complete history was available in 83 patients with VaIN (VaIN2/3, 37.3%; VaIN1, 62.7%) after hysterectomy.  Table 2 shows the indications for previous hysterectomy in patients with VaIN. Among these, 86.7% underwent hysterectomy for cervical lesions, including cervical cancer (30.1%) and precancer (56.6%); 13.3% underwent hysterectomy for noncervical lesions, including uterine fibroid, endometrial cancer, ovarian cancer, and fallopian tube cancer.

We used Thinprep 2000 (TCT) or AutoCyte/PrepStain (LCT) for cytology testing, and Hybrid Capture 2 assay (Qiagen, Hilden, Germany) or Cobas 4800 assay (Roche, Penzberg, Germany) for HR-hrHPV testing. Chi-square tests were performed with SPSS 16.0 software (IBM, New York, USA). A *P* value < 0.05 was considered to be statistically significant.

## 3. Results

### 3.1. Classification of 529 Cases of VaIN

Based on a history of hysterectomy and the presence of only VaIN or VaIN concomitant with cervical or vulvar lesions, 529 cases of VaIN were classified as reported in Table 3. The mean age of the 529 patients with VaIN was 46.0 ± 12.6 years (range: 20–79); 16.1% were diagnosed with VaIN2/3 with a mean age of 50.4 ± 12.2 years (range: 20–72) and 83.9% were diagnosed with VaIN1 with a mean age of 45.1 ± 12.6 years (range: 21–79).

Among 435 patients without a history of hysterectomy, 12.0% were diagnosed with VaIN2/3 and 88.0% were diagnosed with VaIN1; 60.5% were diagnosed with only VaIN and 39.5% were diagnosed with VaIN concomitant with cervical or vulvar lesions. Among patients with only VaIN, 9.1% were VaIN2/3 and 90.9% were VaIN1. Among those with concomitant VaIN, 16.3% were VaIN2/3 and 83.7% were VaIN1. In 94 patients with a history of hysterectomy, 35.1% were diagnosed with VaIN2/3 and 64.9% with VaIN1. The ratio of VaIN2/3 among patients with concomitant cervical lesions was higher than that of patients with only VaIN (*P* = 0.04); the ratio of VaIN2/3 among patients after hysterectomy was higher than that of patients without hysterectomy (*P* = 0.00)

### 3.2. Cytology Results of 405 Cases of VaIN

According to the history of hysterectomy and the diagnosis of only or concomitant VaIN, available cytology reports of 405 cases of VaIN were classified as shown in Table 4. Cytology reports of VaIN can include negative diagnoses for intraepithelial lesion or malignancy (NILM), atypical squamous cells of undetermined significance (ASC-US), LSIL, atypical squamous cells that cannot exclude HSIL (ASC-H), or HSIL.

In 326 cases of VaIN without hysterectomy, the sensitivity of cytology was 59.5%; among these, 10.4% were VaIN2/3 and sensitivity of cytology for VaIN2/3 was 58.8%. In 212 cases of only VaIN, sensitivity of cytology was 52.4%. Among these, 8.96% were VaIN2/3 and sensitivity of cytology was 42.1%. In 114 cases of concomitant VaIN, sensitivity of cytology was 72.8%. Among these, 13.2% were VaIN2/3 and sensitivity of cytology was 80.0%. In 79 cases of VaIN after hysterectomy, sensitivity of cytology was 69.6%. Among these, 32.9% were VaIN2/3 and sensitivity of cytology was 80.8%.

### 3.3. Sensitivity of Cytology, hrHPV, and Cytology/hrHPV Cotesting in 529 Patients with VaIN

A total of 405 cytology reports were available; sensitivity of cytology for the diagnosis of VaIN is reported in Table 5. In 425 patients with VaIN and no history of hysterectomy, 326 cytology reports were available and the sensitivity of cytology for VaIN, VaIN1, and VaIN2/3 was 59.5%, 59.6%, and 58.8%, respectively. In 94 patients with VaIN after hysterectomy, 79 cytology reports were available and the sensitivity of cytology for VaIN, VaIN1, and VaIN2/3 was 69.6%, 64.2%, and 80.8%, respectively.

A total of 349 hrHPV reports were available (Table 5). In 425 patients with VaIN and no history of hysterectomy, 276 hrHPV reports were available and the sensitivity of hrHPV for VaIN, VaIN1, and VaIN2/3 was 86.5%, 85.7%, and 93.5%, respectively. In 94 patients with VaIN after hysterectomy, 73 hrHPV reports were available and sensitivity of hrHPV for VaIN, VaIN1, and VaIN2/3 was 90.4%, 89.4%, and 92.3%, respectively.

In 529 patients with VaIN, 326 cytology/hrHPV cotesting reports were available (Table 5). In 425 patients with VaIN and no history of hysterectomy, 260 cotesting reports were available and sensitivity of cotesting for VaIN, VaIN1, and VaIN2/3 was 91.9%, 91.9%, and 92.0%, respectively. In 94 patients with VaIN after hysterectomy, 66 cotesting reports were available and sensitivity of cotesting for VaIN, VaIN1, and VaIN2/3 was 98.5%, 97.7%, and 100.0%, respectively.

## 4. Discussion

Ratio of VaIN2/3 is higher in concomitant VaIN than in only VaIN and higher in patients after hysterectomy than in patients without hysterectomy (Table 3). In our study, 529 patients with VaIN were included and 17.8% had a history of hysterectomy, including 83.9% VaIN1 and 16.1% VaIN2/3. Zhang et al. retrospectively analyzed 152 patients with VaIN, and 39.5% of the patients had a history of hysterectomy [18], including 45.4% VaIN1 and 54.6% VaIN2/3. The reason why the ratio of VaIN2/3 in their study is higher than ours might be because the ratio of posthysterectomy patients in their study was higher than that in ours (*P* = 0.00).

Cytology sensitivity was higher in VaIN after hysterectomy than in only VaIN without hysterectomy and higher in concomitant VaIN than in only VaIN without hysterectomy (Table 4). The rates of both VaIN and VaIN2/3 after hysterectomy were higher than in only VaIN and VaIN2/3 without hysterectomy (*P* = 0.01), respectively, which demonstrates that the sensitivity of cytology for VaIN after hysterectomy was higher than the sensitivity of cytology for only VaIN and similar to that for concomitant VaIN without hysterectomy. In patients without hysterectomy, the sensitivity of cytology was higher for concomitant VaIN, VaIN2/3, and VaIN1 than for only VaIN, VaIN2/3, and VaIN1 (*P* = 0.00, *P* = 0.03, and *P* = 0.00), respectively. In our view, cytology sampling of VaIN concomitant with cervical lesions includes abnormal cervical cells in addition to abnormal vaginal cells, and cervical cells make up the largest component of exfoliative cells. Thus, their sensitivity of cytology is higher for concomitant VaIN than isolated VaIN. For women after hysterectomy, all cytology samples come from the vagina, and thus the sensitivity of cytology for VaIN was higher than that of isolated VaIN in patients without hysterectomy.

Cytology and hrHPV sensitivity for VaIN were noninferior to sensitivity for CIN2+ (CIN2 or worse) and might be higher in women after hysterectomy. Cytology and hrHPV tests are used to screen cervical lesions. Our study showed that both of these methods can be used to screen for VaIN, especially among women after hysterectomy, because they showed noninferior sensitivity in comparison to women without hysterectomy. In the ATHENA trial, the sensitivity of cytology for CIN2+ was 40.6% (95% confidence interval [CI]: 36.1–45.1%) in women ≥ 25 years of age [19]; liquid-based cytology specimens from 46,887 eligible women ≥ 21 years of age were evaluated at four large regional US laboratories, and there were considerable differences among the laboratories both in the overall rates of cytological abnormalities, ranging from 3.8 to 9.9%, and in the sensitivity of cytology to detect CIN2+, from 42.0 to 73.0%. In contrast, the hrHPV positivity rate varied only from 10.9 to 13.4%, and the sensitivity of hrHPV testing varied from 88.2 to 90.1% [20]. In another study, cytology sensitivity for CIN2+ was 39.5% (95% CI: 29.4–49.5%), Hybrid Capture 2 (HC2) hrHPV sensitivity for CIN2+ was 93.2% (95% CI: 87.1–99.2%), and Aptima hrHPV sensitivity for CIN2+ was 87.8% (95% CI: 80.2–95.5%) [21]. In our study, cytology sensitivity for VaIN2/3 was 58.8–80.8% and hrHPV sensitivity for VaIN2/3 was 92.3–93.5%, whether there was a history of hysterectomy or not, which showed that the sensitivity of cytology and hrHPV for VaIN was noninferior to the sensitivity for CIN2+, and these methods might be used to detect VaIN2/3 and recurrence of cervical cancer in the vaginal apex.

Vaginal or vulvar lesions could be more severe than cervical lesions. In concomitant VaIN, cervical lesions (two locations of the lower genital tract) represented 96.6%, vulvar lesions (two locations) 1.0%, and cervical and vulvar lesions (all three locations) 2.4% of VaIN (Table 1). Generally, the cervix is regarded as the most susceptible and severest location for intraepithelial neoplasia of the lower genital tract, which results in potential neglect in the evaluation of the vagina and vulva during colposcopy. In fact, the most severe lesions can be located in the vagina and vulva rather than the cervix. Therefore, careful examination of the entire lower genital tract in colposcopy is essential for the diagnosis of vaginal and vulvar lesions.

Approximately one-tenth of VaIN2/3 occurred after hysterectomy for noncervical diseases. Our data show that 13.3% of patients with VaIN and 9.7% of patients with VaIN2/3 had a history of hysterectomy for noncervical diseases. We believe that the actual ratio might be higher because many women stop cytology or hrHPV testing, according to the current guideline. Since there might be insufficient assessment of cervical lesions before hysterectomy and patients might be infected with hrHPV before or after hysterectomy, regular cytology or hrHPV testing can help detect VaIN2/3 and vaginal cancer in posthysterectomy patients. The available literature was too limited to develop evidence based recommendations for managing abnormal vaginal cytology and hrHPV screening tests. An algorithm based on expert opinion is proposed for managing women with abnormal vaginal test results [17].

## 5. Conclusion

The sensitivity of cytology and hrHPV for VaIN is noninferior to that of CIN2+, and thus these methods can help in the early detection of VaIN effectively.

## Figures and Tables

**Table 1:** Original composition of vaginal, cervical, and vulvar lesions when history was considered.

Vagina	Cervix	Vulva	Number
HSIL	/	/	18
HSIL	CA	/	14
HSIL	HSIL	/	35
HSIL	LSIL	/	12
HSIL	LSIL	HSIL	1
HSIL	/	HSIL	2
LSIL	/	/	204
LSIL	CA	/	14
LSIL	HSIL	/	56
LSIL	LSIL	/	154
LSIL	LSIL	CA	1
LSIL	LSIL	HSIL	5
LSIL	/	HSIL	1
Total			517

HSIL: high-grade squamous intraepithelial lesion; LSIL: low-grade squamous intraepithelial lesion; / stands for no lesion.

**Table 2:** Indications and rates of previous hysterectomy in 83 VaIN patients.

Indications of hysterectomy	VaIN		VaIN2/3		VaIN1	
	Number	%	Number	%	Number	%
Cervical lesions	72	86.7%	28	90.3%	44	84.6%
Cervical cancer	25	30.1%	11	35.5%	14	26.9%
Cervical precancer	47	56.6%	17	54.8%	30	57.7%
Noncervical lesions	11	13.3%	3	9.7%	8	15.4%
Endometrial cancer	2	2.4%	2	6.5%	0	0.0%
Fallopian tube cancer	1	1.2%	0	0.0%	1	1.9%
Ovarian cancer	1	1.2%	0	0.0%	1	1.9%
Myoma	7	8.4%	1	3.2%	6	11.5%
Total	83	100.0%	31	100.0%	52	100.0%

VaIN: vaginal intraepithelial neoplasia.

**Table 3:** VaIN diagnosed in patients after hysterectomy and with no hysterectomy.

VaIN	Number	Rate
No hysterectomy	435	100.0%
VaIN2/3	52/435	12.0%
VaIN1	383/435	88.0%

Only vaginal lesions	263/435	60.5%
VaIN2/3	24/263	9.1%
VaIN1	239/263	90.9%

Concomitant lesions	172/435	39.5%
VaIN2/3	28/172	16.3%
VaIN1	144/172	83.7%

After hysterectomy	94	100.0%
VaIN2/3	33/94	35.1%
VaIN1	61/94	64.9%

VaIN: vaginal intraepithelial neoplasia.

**Table 4:** Detailed cytology results of VaIN diagnosed in patients after hysterectomy and with no hysterectomy.

VaIN	Cytology number (%)						Sensitivity (%)
	NILM	ASC-US	LSIL	ASC-H	HSIL	CA	
VaIN with uterus	132 (40.5%)	71 (21.8%)	108 (33.1%)	6 (1.8%)	8 (2.5%)	1 (0.3%)	59.5%
VaIN2/3	14 (41.2%)	7 (20.6%)	10 (29.4%)	1 (2.9%)	1 (2.9%)	1 (2.9%)	58.8%
VaIN1	118 (40.4%)	64 (21.9%)	98 (33.6%)	5 (1.7%)	7 (2.4%)	0 (0.0%)	59.6%

Only VaIN	101 (47.6%)	50 (23.6%)	56 (26.4%)	3 (1.4%)	2 (0.9%)	0 (0.0%)	52.4%
VaIN2/3	11 (57.9%)	4 (21.1%)	3 (15.8%)	1 (5.3%)	0 (0.0%)	0 (0.0%)	42.1%
VaIN1	90 (46.6%)	46 (23.8%)	53 (27.5%)	2 (1.0%)	2 (1.0%)	0 (0.0%)	53.4%

Concomitant VaIN	31 (27.2%)	21 (18.4%)	52 (45.6%)	3 (2.6%)	6 (5.3%)	1 (0.9%)	72.8%
VaIN2/3	3 (20.0%)	3 (20.0%)	7 (46.7%)	0 (0.0%)	1 (6.7%)	1 (6.7%)	80.0%
VaIN1	28 (28.3%)	18 (18.2%)	45 (45.5%)	3 (3.0%)	5 (5.1%)	0 (0.0%)	71.7%

VaIN after hysterectomy	24 (30.4%)	16 (20.3%)	24 (30.4%)	4 (5.1%)	11 (13.9%)	0 (0.0%)	69.6%
VaIN2/3	5 (19.2%)	3 (11.5%)	3 (11.5%)	4 (15.4%)	11 (42.3%)	0 (0.0%)	80.8%
VaIN1	19 (35.8%)	13 (24.5%)	21 (39.6%)	0 (0.0%)	0 (0.0%)	0 (0.0%)	64.2%

VaIN: vaginal intraepithelial neoplasia; NILM: negative for intraepithelial lesion or malignancy; ASCUS: atypical squamous cells of undetermined significance; LSIL: low-grade squamous intraepithelial lesion; ASC-H: atypical squamous cells cannot exclude HSIL; HSIL: high-grade squamous intraepithelial lesion; CA: cancer.

**Table 5:** Cytology/hrHPV sensitivity in VaIN patients after hysterectomy and with no hysterectomy.

VaIN	No hysterectomy	After hysterectomy	*P* value
Cytology sensitivity			
VaIN	59.5% (194/326)	69.6% (55/79)	0.098
VaIN1	59.6% (174/292)	64.2% (34/53)	0.532
VaIN2/3	58.8% (20/34)	80.8% (21/26)	0.070
hrHPV sensitivity			
VaIN	86.5% (239/276)	90.4% (66/73)	0.382
VaIN1	85.7% (210/245)	89.4% (42/47)	0.505
VaIN2/3	93.5% (29/31)	92.3% (24/26)	0.855
Cotesting sensitivity			
VaIN	91.9% (239/260)	98.5% (65/66)	0.058
VaIN1	91.9% (216/235)	97.7% (43/44)	0.170
VaIN2/3	92.0% (23/25)	100.0% (22/22)	0.175

VaIN: vaginal intraepithelial neoplasia; hrHPV: high-risk human papillomavirus.
